# New experimental evidence to support roaming in the reaction Cl + isobutene (*i*-C_4_H_8_)

**DOI:** 10.1038/srep40105

**Published:** 2017-01-12

**Authors:** Li-Wei Chen, Ching-Ming Hung, Hiroyuki Matsui, Yuan-Pern Lee

**Affiliations:** 1 Department of Applied Chemistry and Institute of Molecular Science, National Chiao Tung University, 1001 Ta-Hsueh Rd., Hsinchu 30010, Taiwan; 2 Institute of Atomic and Molecular Sciences, Academia Sinica, Taipei 10617, Taiwan

## Abstract

The reaction Cl + isobutene (i-C_4_H_8_) was reported by Suits *et al*. to proceed via, in addition to abstraction, an addition-elimination path following a roaming excursion of Cl; a near-zero translational energy release and an isotropic angular distribution observed at a small collision energy characterized this mechanism. We employed a new experimental method to further characterize this roaming mechanism through observation of the internal distribution of HCl (*v, J*) and their temporal behavior upon irradiation of a mixture of Cl_2_C_2_O_2_ and *i*-C_4_H_8_ in He or Ar buffer gas. With 1–3 Torr buffer gas added to approach the condition of small collision energy, the intensities of emission of HCl (*v* = 1, 2) and the HCl production rates increased significantly; Ar shows a more significant effect than He because Ar quenches Cl more efficiently to reduce the collisional energy and facilitate the roaming path. According to kinetic modeling, the rate of addition-elimination (roaming) increased from *k*_E_ ≈ 2 × 10^5^ s^−1^ when little buffer gas was present to ~1.9 × 10^6^ s^−1^ when 2–3 Torr of Ar was added, and the branching ratio for formation of [HCl (*v* = 2)]/[HCl (*v* = 1)] increased from 0.02 ± 0.01 for abstraction to 0.06 ± 0.01 for roaming.

The reactions of atomic chlorine (Cl) with alkenes (C_*n*_H_2*n*_) play important roles in the chemistry of the troposphere^1^,^2^,^3^,^4^,^5^. These reactions are also of fundamental importance in understanding the reaction kinetics or dynamics that involve a competition between various paths and a site selectivity. The two most significant channels of the primary Cl + C_*n*_H_2*n*_ reactions are the addition of a Cl atom to the C=C double bond to form a thermally stabilized adduct, chloroalkyl radical (•C_n_H_2n_Cl), and the metathesis reaction to form HCl and an alkyl radical (•C_n_H_2n−1_); the metathesis includes a direct abstraction of a H atom of the alkene by the Cl atom and an elimination of HCl from the energetic adduct •C_n_H_2n_Cl^6^. It is generally accepted that the abstraction dominates at low pressure, whereas the addition-stabilization mechanism becomes more important at high pressure^6^,^7^,^8^.

Employing crossed molecular beams to investigate the dynamics of the reaction Cl + *i*-C_4_H_8_, Suits and coworkers detected C_4_H_7_ with a slice ion-imaging method^9^,^10^. These authors found that the addition-elimination path occurs from an abstraction-like Cl-H-C geometry rather than a conventional three-center or four-center transition state, and this geometry is attained through roaming excursions of the Cl atom from the initially formed adduct. The experimental observations to support this roaming mechanism are limited: a release of translational energy with a maximal distribution near zero energy and a fully isotropic angular distribution of C_4_H_7_ product were observed when a small collisional energy was employed.

Preston *et al*. employed velocity-map imaging to measure the quantum states and velocity of HCl produced from reactions Cl + propene, *i*-C_4_H_8_, and dimethylbutene; signals from both direct abstraction and addition-elimination were observed^11^. These authors simulated trajectories that indicated the importance of a large-amplitude excursion of the Cl atom far from equilibrium geometry within the chloroalkyl complex, which ultimately led to formation of HCl + allyl fragmentation^12^, but a clear distinction in the internal energy distribution of HCl produced via abstraction from that via addition-elimination of Cl + *i*-C_4_H_8_ was unavailable.

Roaming dynamics that involve reactions of a radical (or an atom) with a radical, resulting typically from photofragmentation of a suitable precursor, are now widely recognized as an important path in unimolecular decompositions^13^,^14^,^15^,^16^,^17^, but their roles in radical (or an atom) - molecule reactions remain less certain. Molecular dynamics simulations of the unimolecular dissociation of energetic C_2_H_4_OH radicals reveal a minor roaming channel of OH + C_2_H_4_ that leads to formation of H_2_O and C_2_H_3_^18^. Roaming was observed in photolysis of NO_3_ via both ground- and excited-state surfaces to form NO + O_2_ and supported by theoretical calculations^19^,^20^,^21^,^22^. Both these reactions are initiated from photodissociation of free radicals; reported roaming reaction initiated from reactions of radical (atom) and molecule is rare.

In the roaming mechanism of Cl + *i*-C_4_H_8_, because the reaction to form HCl + C_4_H_7_ has a small exothermicity (~68 kJ mol^−1^) and because the addition-elimination path occurs at the abstraction-like near-linear Cl−H−C geometry, to distinguish the internal distributions of HCl produced via abstraction from that via the addition-roaming-elimination path is difficult. In this work we demonstrate a new experimental method to characterize the roaming path in the atom-molecule reaction Cl + *i*-C_4_H_8_ → HCl + C_4_H_7_ by using a step-scan Fourier-transform infrared (FTIR) spectrometer to obtain time-resolved IR emission spectra of HCl^23^,^24^,^25^.

## Results and Discussion

### Emission spectra of HCl

According to the results of Joalland *et al*.^9^,^10^, roaming of Cl + *i*-C_4_H_8_ was most prominent at a small collisional energy (~17 kJ mol^−1^). Because the Cl atoms produced upon photolysis still have significant kinetic energy, collisional quenching to a nearly thermal distribution of kinetic energy is expected to enhance the roaming path. We hence compared the effect of adding He or Ar up to a total pressure of 3 Torr to quench the kinetic energy of Cl atoms after photolysis.

Similar experiments with the same amount of Cl_2_C_2_O_2_ (10–11 mTorr) and laser fluence were performed for three sets of data; the conditions are listed in Table 1. Set A was for investigation of the dependence on the concentration of *i*-C_4_H_8_ with little buffer gas, set B was for investigation of the effect of adding He as a quencher, and set C was for investigation of the effect of adding Ar as a quencher. The absorption cross section of Cl_2_C_2_O_2_ at 248 nm is ~3.1 × 10^−19^ cm^2^ molecule^−1^ 26. Typically, ~13% of Cl_2_C_2_O_2_ was photodissociated with a fluence of 343 mJ cm^−2^.

**Figure 1:**
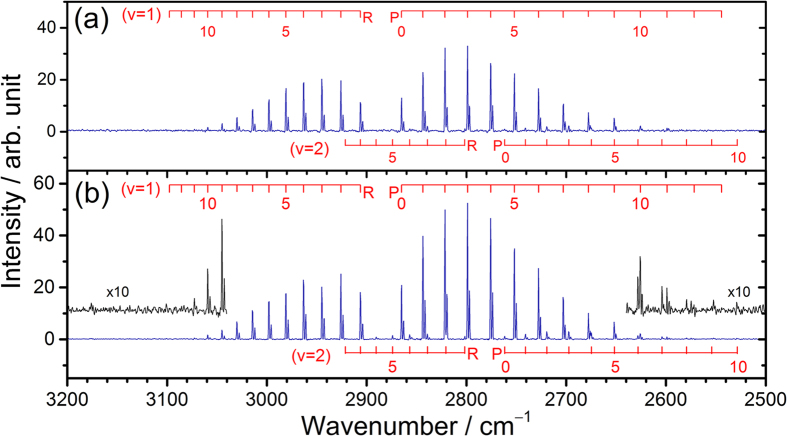
Emission spectra of HCl in spectral region 2500–3200 cm^−1^ recorded 0–5 μs after photolysis at 248 nm. (**a**) A flowing mixture of Cl_2_C_2_O_2_ (10 mTorr), *i*-C_4_H_8_ (213 mTorr) and Ar (10 mTorr). (**b**) A flowing mixture of Cl_2_C_2_O_2_ (11 mTorr), *i*-C_4_H_8_ (226 mTorr) and Ar (2.99 Torr). Spectral resolution is 0.7 cm^−1^. The assignments are shown as sticks; numbers indicate rotational quantum number *J*′.

Emission spectra of HCl at resolution 0.7 cm^−1^ recorded 0–5 μs after photolysis of Cl_2_C_2_O_2_ (~11 mTorr), *i*-C_4_H_8_ (~220 mTorr), and Ar (0.010 and 2.99 Torr) are presented in Fig. 1(a,b), respectively; lines of H^35^Cl and H^37^Cl are well resolved. The vibration-rotational assignments of each line based on spectral parameters reported by Arunan *et al*.^27^ and Coxon and Roychowdhury^28^ are shown as sticks for transitions of *v*′ = 1, *J*′ ≤ 13 and *v*′ = 2, *J*′ ≤ 10. At ~3.2 Torr, formation of the HCl (*v* = 2) was enhanced slightly, as shown in Fig. 1(b). For comparison, Preston *et al*. observed *v*′ = 1, *J*′ ≤ 7 and *v*′ = 2, *J*′ = 1 in their jet experiments using velocity-map ion imaging^11^. The energetics, discussed in Supplementary Sec. A, indicate that the available energy for formation of HCl + C_4_H_7_ is ~81 kJ mol^−1^ (6770 cm^−1^) when Cl atoms are thermalized by collisions with Ar or He; this energy is close to the energy of HCl (*v* = 2, *J* = 10) at 6743 cm^−1^.

### Rotational temperature

The rotational distributions of HCl are Boltzmann, as shown in Supplementary Fig. S1 for spectra recorded 0–1 μs after photolysis. Derivation of nascent rotational temperature is discussed in Supplementary Sec. B. Nascent rotational temperatures and average rotational energies observed in three sets of experiments are listed in Supplementary Table S1. In all cases, the rotational temperatures are similar (deviations within 15%) and decrease slightly from ~370 K to ~340 K as the pressure increases to ~3.2 Torr; detailed data are in Supplementary Table S1. The average rotational energy is hence ~3 kJ mol^−1^, independent of pressure. The small rotational energy indicates that, if roaming occurs more significantly at greater pressure, the HCl product generated from this path has rotational excitation similar to that from abstraction. This condition is consistent with a prediction that the roaming (addition-elimination) path occurs from the abstraction-like near-linear Cl-H-C geometry^9^.

### Vibrational excitation and relative intensity of HCl

Because only HCl (*v* = 1) and HCl (*v* = 2) were observed, to determine accurately the vibrational temperature and energy is difficult. We list *P*_v=2_/*P*_v=1_ and the relative intensity *y* of HCl (*v* = 1 and 2) in various experiments in Table 1; the relative intensity was compared to the results of *P*_T_ = 3.23 Torr (Ar) in set C. When little buffer gas was present, [HCl (*v* = 2)]/[HCl (*v* = 1)] = 0.015 ± 0.004, whereas when ~3 Torr of Ar was added, [HCl (*v* = 2)]/[HCl (*v* = 1)] = 0.045 ± 0.001. Because of the excellent ratio of signal to noise in the spectra, the error in the determination of relative population was estimated to be less than 25% of the value when the signal is small; the difference in these ratios is hence significant, as can also be seen from the consistency in ratios determined in various experiments (Table 1). The small difference is partly because the abstraction path still contributes when the buffer gas was added and partly because the difference in [HCl (*v* = 2)]/[HCl (*v* = 1)] between abstraction and addition-elimination is not so large. Nevertheless, the vibrational excitation of HCl was clearly enhanced when the buffer gas was added.

Because we could not measure the population of HCl (*v* = 0), these relative intensities consequently provide only a rough estimate of the extent of HCl produced. Nevertheless, it is clear from Table 1 that the intensity of (vibrationally excited) HCl was enhanced by as much as 16 times when a buffer gas was added, and adding Ar showed a greater enhancement than adding He.

### Rate coefficient derived from kinetic fitting of vibrational temporal profiles

**Figure 2:**
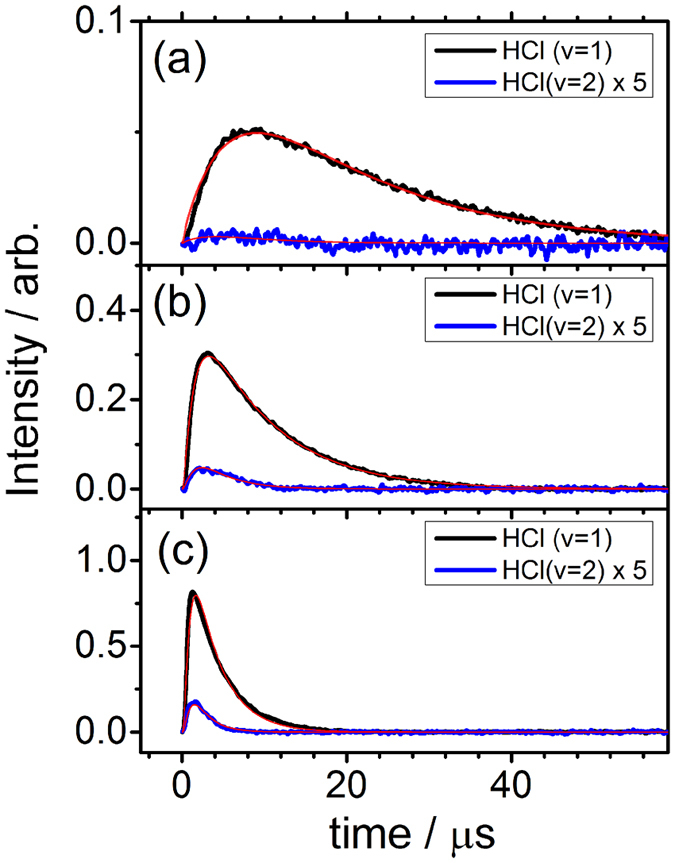
Comparison of observed temporal profiles of HCl (*v*) with simulations. (**a**) A flowing mixture of Cl_2_C_2_O_2_ (10 mTorr), *i*-C_4_H_8_ (213 mTorr) and Ar (10 mTorr). (**b**) A flowing mixture of Cl_2_C_2_O_2_ (10 mTorr), *i*-C_4_H_8_ (226 mTorr) and He (2.99 Torr). (**c**) A flowing mixture of Cl_2_C_2_O_2_ (10 mTorr), *i*-C_4_H_8_ (226 mTorr) and Ar (2.99 Torr). HCl (*v* = 1) is in black and HCl (*v* = 2), multiplied by a factor of 5, is in blue. The kinetic simulations are in red.

Because the rotational temperature of HCl is near 360 K and independent of pressure, the rotational temporal profile provides little information about the kinetics of the reaction. We thus analyzed only the vibrational temporal profiles; some representative plots in experimental set A for *P*_T_ = 0.23 Torr, set B for *P*_T_ = 3.23 Torr (He), and set C for *P*_T_ = 3.23 Torr (Ar) are shown in Fig. 2. Temporal profiles for other experiments in sets A–C are presented in Supplementary Figs S2–S4, respectively.

**Figure 3:**
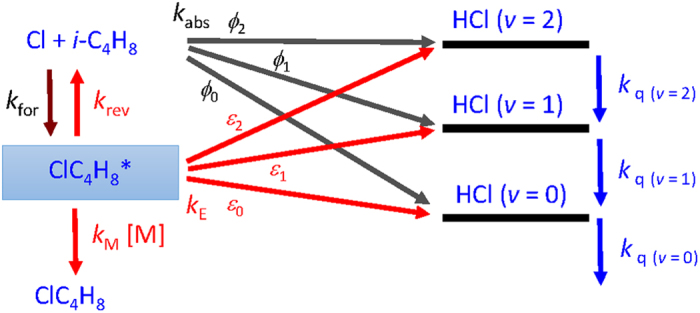
Reaction mechanism of Cl + *i*-C_4_H_8_.

We consider the reaction mechanism depicted in Fig. 3. The reaction of Cl + *i*-C_4_H_8_ forms energetic adduct ClC_4_H_8_* with a rate coefficient *k*_for_; ClC_4_H_8_* might be stabilized by collision with the third-body *M* (rate coefficient *k*_M_ [M]), dissociate back to the reactants (rate coefficient *k*_rev_), or eliminate HCl via roaming-elimination (rate coefficient *k*_E_). The abstraction and elimination channels might produce HCl with distinct vibrational distributions. The abstraction reaction (rate coefficient *k*_abs_) has three channels to produce HCl (*v* = 0), HCl (*v* = 1), and HCl (*v* = 2) with branching ratios *ϕ*_0_, *ϕ*_1_, and *ϕ*_2_, respectively. The elimination reaction has, similarly, three channels with branching ratios *ε*_0_, *ε*_1_, and *ε*_2_ for production of HCl (*v* = 0–2), respectively. The decay rate coefficients *k*_q (*v*)_ are also separated for each vibrational level of HCl and include both quenching and loss. The solution of [HCl(*t*)] contains three exponential terms, but they are complex functions of rate coefficients, as discussed in Supplementary Sec. C. Hence, instead of fitting the temporal profiles to three exponential terms, we simulated the temporal profile with given rate coefficients and compare with experimental results. We systematically varied the rate coefficients using mathematical tools to derive the best fit between the experimental data and the simulated temporal profiles with the least deviations.

Because eight rate coefficients are involved in this mechanism, it is unlikely to derive a unique fit of these parameters from the temporal profiles. We thus fixed some well-known rate coefficients and varied only *k*_E_ and *k*_rev_, and the vibrational branching ratios of abstraction (*ϕ*_2_/*ϕ*_1_) and elimination (*ε*_2_/*ε*_1_) reactions. The total rate coefficient *k*_T_ for reaction Cl + *i*-C_4_H_8_ at 1 bar (air) was determined to be *k*_T_ = (3.40±0.28)×10^−10^ cm^3^ molecule^−1^ s^−1^ by Ezell *et al*.^29^. Following the empirical additivity rules based on data of reactions of Cl with alkanes and alkenes, these authors proposed that *k*_abs_ = 6.8×10^−11^ cm^3^ molecule^−1^ s^−1^ for the formation of HCl and *k*_add_ = 2.7×10^−10^ cm^3^ molecule^−1^ s^−1^ for the formation of stabilized ClC_4_H_8_; the value of *k*_abs_ should be *k*_meta_, rate coefficient of metathesis, that includes abstraction and addition-elimination. Considering that abstraction reactions occur only on the methyl groups of propene and isobutene, one estimates *k*_abs_ = 4.6×10^−11^ cm^3^ molecule^−1^ s^−1^ because *i*-C_4_H_8_ has two methyl moieties and *k*_abs_ was determined to be 2.3×10^−11^ cm^3^ molecule^−1^ s^−1^ for Cl + propene^7^. The value of *k*_for_ for the reaction Cl + *i*-C_4_H_8_ can be taken as the difference between total rate coefficient *k*_T_ and *k*_abs_, that is, *k*_for_ = 2.94×10^−10^ cm^3^ molecule^−1^ s^−1^, which corresponds satisfactorily with the value for Cl + propene, *k*_∞_ = (2.7 ± 0.4)×10^−10^ cm^3^ molecule^−1^ s^−1^. The rate coefficient at the low-pressure limit for Cl + *i*-C_4_H_8_ is unreported, but the corresponding value for Cl + propene is *k*_0_ = (4.0 ± 0.4)×10^−28^ cm^6^ molecule^−2^ s^−1^ 7. If we assume that the termolecular reaction Cl + *i*-C_4_H_8_ + M to form ClC_4_H_8_ is still in the linear regime for *P*_T_ ≤ 3 Torr, and use *k*_0_ of the reaction of Cl + propene for Cl + *i*-C_4_H_8_, *k*_M_ = (4.0 ± 0.4)×10^−28^ [*i*-C_4_H_8_] [*M*] cm^6^ moleule^−2^ s^−1^.

**Figure 4:**
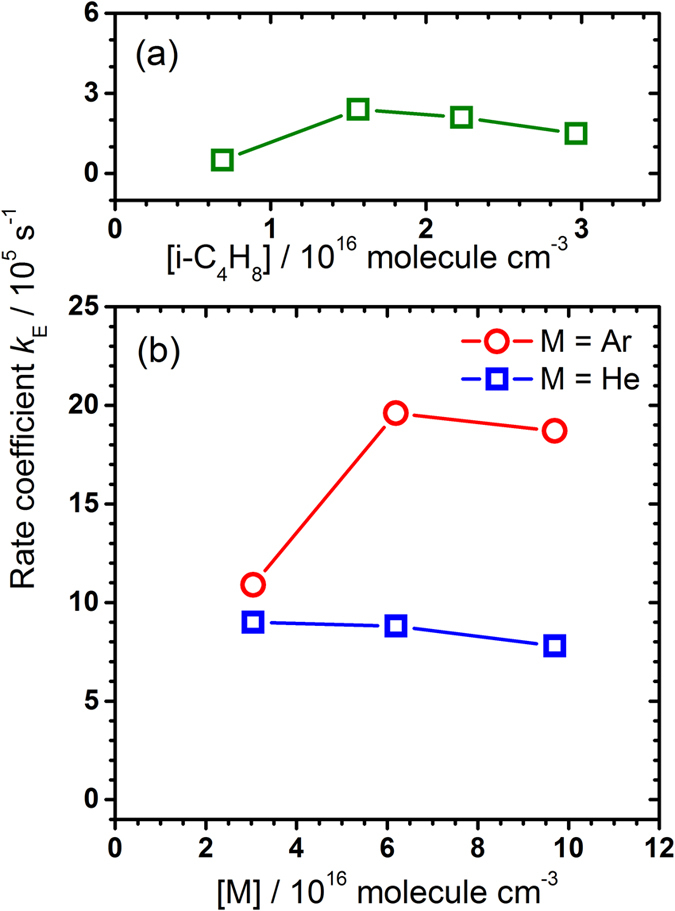
Fitted *k*_E_ in three sets of experiments. (**a**) A flowing mixture of Cl_2_C_2_O_2_ (10 mTorr), Ar (10 mTorr), and *i*-C_4_H_8_ (213–914 mTorr). (**b**) A flowing mixture of Cl_2_C_2_O_2_ (10 mTorr), *i*-C_4_H_8_ (226 mTorr) and buffer gas He (0.94–2.99 Torr, symbol □) or Ar (0.94–2.99 Torr, symbol ○).

Because HCl (*v* = 0) is unobserved in emission, we fit only the temporal profiles of HCl (*v* = 1) and HCl (*v* = 2) simultaneously on varying *k*_q_, *ϕ*_2_/*ϕ*_1_, *k*_E_, and *ε*_2_/*ε*_1_ to derive the best fit between the experimental data and the simulated temporal profiles. In these fits, *k*_abs_ = 4.6×10^−11^ cm^3^ molecule^−1^ s^−1^, *k*_for_ = 2.9×10^−10^ cm^3^ molecule^−1^ s^−1^, *k*_M_ = 4.0×10^−28^ [*i*-C_4_H_8_] [*M*] cm^6^ molecule^−2^ s^−1^ were fixed, and *k*_rev_ + *k*_M_ + *k*_E_ = *k*_for_ [*i*-C_4_H_8_]. The representative fitted temporal profiles of HCl (*v*) for experiments in set A (0.22 Torr), set B (3.23 Torr), and set C (3.23 Torr, Ar) are shown in Fig. 2; additional profiles are shown in Supplementary Figs S2–S4. We plot *k*_E_ as a function of density [*M*] of *i*-C_4_H_8_ or He or Ar in Fig. 4 for experimental sets A–C. Fitted results of *k*_E_, *k*_q_, *ϕ*_2_/*ϕ*_1_, and *ε*_2_/*ε*_1_ are listed in Table 1.

For experiments in set A with varying *i*-C_4_H_8_ and little buffer gas, *k*_E_ remains small and is nearly constant with *k*_E_ = (1.6 ± 0.8) × 10^5^ s^−1^ (Fig. 4(a)). For He as a buffer gas, *k*_E_ is much greater than those without buffer gas and remains nearly constant with *k*_E_ = (8.5 ± 0.7) × 10^5^ s^−1^ for [He] = (3.0–9.7) × 10^16^ molecule cm^−3^, whereas *k*_E_ increases from 1.1 × 10^5^ s^−1^ at [Ar] = 3.0 × 10^16^ molecule cm^−3^ to (1.9 ± 0.1) × 10^6^ s^−1^ at [Ar] = (6.2–9.7) × 10^16^ molecule cm^−3^ (Fig. 4(b)). Such a significant increase in *k*_E_ for experiments with an added quencher, especially Ar, can be explained only with an enhanced addition-elimination channel induced by the collisional quenching of the kinetic energy of Cl; because the mass of Ar is similar to that of Cl, Ar is a much more efficient quencher than He for kinetic energy of Cl.

Detailed sensitivity and error analysis are available in Supplementary Sec. D. The deviations derived in these analysis are much smaller than the enhancement of *k*_E_ observed in experimental sets B and C as compared to those in experiments with little buffer gas.

For experimental sets B and C (in which roaming is more important), *ε*_2_/*ε*_1_ are consistent with average (5.5 ± 0.9) %. We thus fixed *ε*_2_/*ε*_1_ = 0.06 to fit the data once more; a much more consistent value of *ϕ*_2_/*ϕ*_1_ with average (3.0 ± 0.7) % for set A (in which abstraction is more important) was derived, as shown in parentheses in Table 1 (other fitted parameters are listed in Supplementary Table S2). The *ϕ*_2_/*ϕ*_1_ value of 0.02 when little buffer gas was added is the smallest and is taken as the *ϕ*_2_/*ϕ*_1_ value for abstraction. This value is consistent with the average ratio of [HCl(*v* = 2)]/[HCl(*v* = 1)] = 0.022 in experimental set A derived from integrated intensities. The ratio of *ε*_2_/*ε*_1_ = 0.06 ± 0.01 for roaming, derived from kinetic fitting, is greater than values [HCl(*v* = 2)]/[HCl(*v* = 1)] = 0.035–0.045 in experimental set C because the latter values include contributions from both abstraction and roaming.

These results indicate that the roaming path generates more vibrationally excited HCl than the abstraction path, consistent with the expectation according to consideration of the structures of the transition states, because the roaming transition state has an H−Cl distance of 2.37 Å^9^, much greater than the H−Cl distance of 1.59 Å for the transition state of H-abstraction^30^ and the equilibrium distance 1.275 Å of HCl. In the case of photolysis of acetaldehyde, the roaming transition state has a C−H distance of 1.722 Å, much greater than the equilibrium C−H distance of 1.093 Å of CH_4_^31^. The CH_4_ product after roaming of CH_3_ around HCO was found to have extreme significant vibrational excitation, with the vibrational distribution peaked at ~95% of the total available energy^32^. For the reaction of Cl + *i*-C_4_H_8_, the average available energy for formation of HCl + C_4_H_7_ is ~81 kJ mol^−1^ (6770 cm^−1^) when Cl atoms are thermalized by collisions with Ar or He. This energy can populate HCl only up to *v* = 2, *J* = 10, so the extent of vibrational excitation is not as great as that of the roaming of CH_3_ + HCO.

This competition between abstraction and addition-elimination via a long-lived complex is similar to the early work on H + ICl by Polanyi *et al*.^33^. In their 3D trajectory studies, they reported that the HCl formed with a small internal energy resulted from reaction of H directly at the Cl-end of ICl, whereas the HCl formed with high internal energy was produced by migration of H from the I-site to the Cl-site, following a lingering interaction of H with I.

## Conclusion

In summary, experimental evidence of three types supports the involvement of the roaming mechanism that is expected to be enhanced at small collisional energy as more buffer gas is added to thermalize the kinetic energy of Cl after photolysis; Ar is expected to be more effective than He in quenching the kinetic energy of Cl. The evidence follows. (1) The intensity of HCl (*v* = 1 and 2) was enhanced by as much as sixteen times when buffer gas at 1–3 Torr was added, more so when the same amount of Ar than when He was added. (2) The observed temporal profiles indicate a significantly increased rate for the formation of HCl when the buffer gas was added; Ar was more effective than He. According to the kinetic modeling, the rate of addition-elimination (roaming) increased from *k*_E_ ≈ 2 × 10^5^ s^−1^ when little buffer gas was present to ~8.5 × 10^5^ s^−1^ when 1–3 Torr of He was added, and ~1.9 × 10^6^ s^−1^ when 2–3 Torr of Ar was added. (3) Ratio [HCl (*v* = 2)]/[HCl (*v* = 1)] increased when Ar (1–3 Torr) was added relative to when little buffer gas was present. According to the kinetic modeling, we derived a branching ratio *ε*_2_/*ε*_1_ = 0.06 ± 0.01 from roaming and *ϕ*_2_/*ϕ*_1_ = 0.02 ± 0.01 from abstraction. This result is consistent with an expectation that HCl produced from the roaming mechanism to have greater vibrational excitation, even though the excitation is limited by the small exothermicity.

The rotational temperature of HCl near 360 K shows no significant variation under varied pressure, indicating that the rotational excitation of HCl from abstraction and roaming is similarly small, consistent with the proposal that roaming (addition-elimination) occurs from the abstraction-like linear Cl-H-C geometry.

## Methods

The step-scan Fourier-transform infrared (FTIR) spectrometer coupled with a set of Welsh mirrors to obtain time-resolved IR emission spectra has been described^23^,^24^,^25^. A gaseous flowing mixture of isobutene and oxalyl chloride (Cl_2_C_2_O_2_) was irradiated with an excimer laser at 248 nm for production of Cl to initiate the reaction of Cl + i-C_4_H_8_. We used Cl_2_C_2_O_2_ instead of Cl_2_ as a source of Cl atoms because the secondary reactions of C_4_H_7_ with Cl_2_ might interfere.

The sizes of the photolysis beams at the detection center were ~11.0 × 5.7 mm^2^ with a fluence 345−380 mJ cm^−2^ from a KrF laser (Coherent, COMPexPro-50) at 248 nm. The transient signal detected with an InSb detector (rise time 0.22 μs) was further amplified 20‒160 times (bandwidth 1 MHz) before being digitized and recorded with an external data-acquisition board (12-bit) at resolution 25 ns. For survey spectra, data were typically averaged over 60 laser pulses at each scan step; 1332 scan steps were performed to yield an interferogram resulting in a spectrum in a region 1800–7800 cm^−1^ at resolution 12 cm^−1^. To detect emission of HCl, we used undersampling with two IR filters (Spectrogon SP-4300 and OCLI W03999-4) to allow passage of light in the region 2350−3250 cm^−1^. Data were typically averaged over 30 laser pulses at each scan step; 3578 scan steps were performed to yield an interferogram resulting in a spectrum of resolution 0.7 cm^−1^. To improve the ratio of signal to noise (S/N) of the spectra of HCl, four spectra recorded under nearly the same experimental conditions were averaged. To improve further the S/N ratio, n consecutive time-resolved spectra were summed to yield spectra representing emission at intervals of *n* × 25 ns; typically *n* = 40 and spectra at 1-μs intervals were used.

Samples of Cl_2_C_2_O_2_ and Ar (or He) were injected into the vacuum chamber as a diffusive beam through a slit-shaped inlet. The *i*-C_4_H_8_ sample has vapor pressure ~340 Torr at 298 K. Additional He or Ar in a minimal pressure (~10 mTorr) was added near the entrance of the photolysis port to suppress the formation of a solid deposit on the quartz window. The partial pressures of each species were calculated by the flow rates of each species, the total flow rate, and the total pressure.

Cl_2_C_2_O_2_ (>98%, Lancaster) and *i*-C_4_H_8_ (99%, Sigma-Aldrich) were purified using the freeze-pump-thaw method. Ar (Specialty Gases of America, 99.9995%) and He (Specialty Gases of America, 99.9995%) were used as received.

## Additional Information

**How to cite this article**: Chen, L.-W. *et al*. New experimental evidence to support roaming in the reaction Cl + isobutene (*i*-C_4_H_8_). *Sci. Rep.* **7**, 40105; doi: 10.1038/srep40105 (2017).

**Publisher's note:** Springer Nature remains neutral with regard to jurisdictional claims in published maps and institutional affiliations.

## Supplementary Material

Supplementary Material

## Figures and Tables

**Table 1:** Experimental conditions, relative intensities *y* of HCl, observed ratios of [HCl(*v* = 2)]/[HCl(*v* = 1)], fitted rate coefficients of elimination *k*_E_ and their associated *k*_rev_, rate coefficient of quenching *k*_q_, branching ratios for formation of HCl (*v* = 2) over HCl (*v* = 1) *ϕ*_2_/*ϕ*_1_ (from abstraction) and *ε*_2_/*ε*_1_ (from elimination) in three sets of experiments.

Set	Conditions^a^		Experiment		Kinetic fit of HCl (*v* = 1) and HCl (*v* = 2)				
	*P*_i-C4H8_/Torr	*P*_M_/Torr	*y*^b^	[HCl(*v* = 2)]/[HCl(*v* = 1)]	*k*_E_/10^5^ s^−1^	*k*_rev_/10^5^ s^−1^	*k*_q_/10^5^ s^−1^	*ϕ*_2_/*ϕ*_1_ (abs.)	*ε*_2_/*ε*_1_ (elim.)
A	0.213	0.010 (Ar)	0.06	0.015 ± 0.004	0.5	19.8	0.6	0.00 (0.02)^c^	0.23
	0.482	0.010 (Ar)	0.20	0.024 ± 0.004	2.4	43.5	1.0	0.05 (0.03)	0.00
	0.687	0.011 (Ar)	0.17	0.023 ± 0.005	2.1	63.4	1.5	0.01 (0.03)	0.20
	0.914	0.011 (Ar)	0.16	0.026 ± 0.007	1.5	85.6	2.1	0.05 (0.04)	0.00
B	0.224	0.940 (He)	0.57	0.035 ± 0.002	9.0	11.4	1.0	0.04 (0.02)	0.04
	0.225	1.910 (He)	0.47	0.034 ± 0.002	8.8	10.9	1.1	0.00 (0.02)	0.06
	0.226	2.990 (He)	0.37	0.031 ± 0.002	7.8	10.9	1.2	0.00 (0.01)	0.06
C	0.224	0.940 (Ar)	0.75	0.035 ± 0.002	10.9	9.5	1.2	0.00 (0.00)	0.05
	0.225	1.910 (Ar)	0.72	0.038 ± 0.001	19.6	0.0	2.1	0.00 (0.00)	0.05
	0.226	2.990 (Ar)	1.00	0.045 ± 0.001	18.7	0.0	3.1	0.01 (0.05)	0.07

^a^Partial pressure of Cl_2_C_2_O_2_ is 10–11 mTorr.

^b^Intensity of HCl relative to that from the experiment with *P*_Ar_ = 2.99 Torr.

^c^Fitted results with *ε*_2_/*ε*_1_ fixed at 0.05 are listed in parentheses.
