# Supplementary material for: New experimental evidence to support roaming in the reaction Cl + isobutene (i-C4H8)
Source: Sci Rep. 2017 Jan 12;7:40105. doi: 10.1038/srep40105 (PMC5228202; doi:10.1038/srep40105)
Supplement: Supplementary Material [file srep40105-s1.pdf]

Supplementary Information

New experimental evidence to support roaming in the reaction Cl + isobutene (*i*-C_4H_8)

Li-Wei Chen[1], Ching-Ming Hung[1], Hiroyuki Matsui[1], and Yuan-Pern Lee[1,2]*

[1]Department of Applied Chemistry and Institute of Molecular Science, National Chiao Tung University, 1001 Ta-Hsueh Rd., Hsinchu 30010, Taiwan, [2]Institute of Atomic and Molecular Sciences, Academia Sinica, Taipei 10617, Taiwan. *E-mail: yplee@mail.nctu.edu.tw (YPL)

***Corresponding Author**

E-mail: yplee@mail.nctu.edu.tw

Index

A. Energetics 1

B. Rotational temperature and energy 1

C. Solution of the differential equation for HCl(*t*) 3

D. Sensitivity and error analyses 4

Table S1. Experimental conditions, nascent rotational temperatures T_R for HCl (v = 1) and HCl (v = 2), average rotational energies (E_R) observed in three sets of experiments. 6

Table S2. Experimental conditions, fitted rate coefficients of elimination k_E and their associated k_{rev}, rate coefficient of quenching k_q, branching ratios for formation of HCl (v = 2) over HCl (v = 1) ϕ_2/ϕ_1 (from abstraction) with $\varepsilon_2/\varepsilon_1$ (from elimination) fixed at 0.06 in three sets of experiments. 7

Figure S1. Semi-logarithmic plots of relative rotational populations of HCl (v = 1 and 2) 0–1 μs after photolysis. 8

Figure S2. Temporal profiles of HCl (v = 1) and HCl (v = 2) in experimental set A with a flowing mixture of $Cl_2C_2O_2$ (10 mTorr), *i*-C_4H_8 (213−914 mTorr) and Ar (10 mTorr). 9

Figure S3. Temporal profiles of HCl (v = 1) and HCl (v = 2) in experimental set B with a flowing mixture of $Cl_2C_2O_2$ (10 mTorr), *i*-C_4H_8 (226 mTorr) and He (0.94−2.99 Torr). 10

Figure S4. Temporal profiles of HCl (v = 1) and HCl (v = 2) in experimental set C with a flowing mixture of $Cl_2C_2O_2$ (10 mTorr), *i*-C_4H_8 (226 mTorr) and Ar (0.94−2.99 Torr). 11

Figure S5. Sensitivity analysis. 12

Figure S6. Comparison of observed temporal profiles of HCl (v) with simulations using k_E = 0. 13

A. Energetics

Suits and coworkers employed the CBS-QB3 method to predict that an abstraction of the H atom at the allylic site of *i*-C_4H_8 by Cl to form HCl and (•CH_2)(CH_3)C=CH_2, is exoergic by 67.8 kJ mol^{-1}, whereas abstraction at the vinyl site with coproduct $H\dot{C}$=C(CH_3)$_2$ is unlikely to occur because it is endoergic by 30.5 kJ mol^{-1}.[1, 2] Adducts 1-chloro-2-methyl-2-propyl [or chloro-*t*-butyl, •C(CH_3)$_2$$CH_2$Cl] and 2-chloro-2-methylpropyl [•CH_2C(CH_3)$_2$Cl] are formed without a barrier, with exothermicities 92.5 and 84.3 kJ mol^{-1}, respectively. The former was predicted to be 89.7 kJ mol^{-1} by Chu *et al.* who employed the MP2/aug-cc-pVTZ method.[3]

When $Cl_2C_2O_2$ was photodissociated at 248 nm (corresponding to 482 kJ mol^{-1}), the Cl atom was reported to have average translational energy of 47.2 kJ mol^{-1} for the first dissociated Cl atom and 14.5 kJ mol^{-1} for the second dissociated Cl atom.[4] The reaction of Cl + *i*-C_4H_8 $\rightarrow$ HCl + C_4H_7 is exothermic by ~68 kJ mol^{-1}.[1] Considering the internal energy of *i*-C_4H_8 ~7.9 kJ mol^{-1} at 298 K, we expect that the available energy for HCl + C_4H_7 would be ~123 kJ mol^{-1} (10283 cm^{-1}) under collisionless conditions and ~81 kJ mol^{-1} (6770 cm^{-1}) when the Cl atom is thermalized with collisions with Ar or He; these two values serve as upper and lower limits of average available energy in this reaction. The highest level of HCl observed in our experiments was v = 2 and J = 10 at 6743 cm^{-1}, corresponding to energy of 80.6 kJ mol^{-1} above the ground state.

B. Rotational temperature and energy

Each vibration-rotational line in the *P*-and *R*-branches was corrected for the instrument-response function, and divided by its respective Einstein coefficient[5] to yield a relative population $P_v(J)$, in which v and J represent vibrational and rotational quantum numbers of the upper states.

Semi-logarithmic plots of population $P_v(J)/(2J + 1)$ versus E_{rot} (in cm^{-1}) for HCl (v =

1, 2), derived from the spectrum recorded in the range 0−1 μs, are shown in Fig. S1 for experiments at total pressures P_T = 0.23 and 3.23 Torr described previously. Each plot was fitted to a linear function to yield a rotational temperature T_R. Derived T_R are 416±43 K (v = 1) for P_T = 0.23 Torr, and 346±41 K (v = 1) and 347±76 K (v = 2) for P_T = 3.23 Torr; error limits listed represent one standard deviation in fitting. For comparison, Preston *et al*. reported T_R = 210 K at v = 1 in their velocity-map ion imaging experiments.[6]

Average rotational energies were E_{rot} = 3.5±0.3 kJ mol^{-1} (v = 1) and E_{rot} = 2.1±3.9 kJ mol^{-1} (v = 2) for P_T = 0.23 Torr, and E_{rot} = 2.9±0.2 kJ mol^{-1} (v = 1) and E_{rot} = 2.4±0.3 kJ mol^{-1} (v = 2) for P_T = 3.23 Torr; the rotational energy of v = 2 was smaller than that of v = 1 because much less rotational levels were observed, even though the rotational temperatures are similar. The rotational energy of level v = 0 cannot be measured in our emission experiments.

The variation of rotational temperature as a function of time is small. With a short extrapolation from these data according to an exponential decay of temperature to 298 K, we estimated the nascent rotational temperature to be 420 ± 40 K for HCl (v = 1) at P_T = 0.23 Torr, and 350 ± 40 K for HCl (v = 1) at P_T = 3.23 Torr (Ar), respectively; the average ratios of the nascent rotational temperature to that determined at period 0–1 μs are 1.00 ± 0.14 and 1.02 ± 0.17, respectively. The nascent rotational energies are hence similar to the values derived for period 0−1 μs, as listed in Table S1; the rotational temperature of HCl (v = 2) makes a negligible contribution to the rotational energy because of its small population. In all cases, the rotational temperatures are similar (deviations within 15 %) and decrease slightly to ~340 K as the pressure increases to ~3.2 Torr. The average rotational energy is hence ~3 kJ mol^{-1}, independent of pressure.

C. Solution of the differential equation for HCl(*t*)

The mechanism of the reactions of Cl + i-C_4H_8 is expected to follow these reactions,

$Cl + i\text{-}C_4H_8 \rightarrow HCl + C_4H_7$	k_{abs}	(1)
$Cl + i\text{-}C_4H_8 \rightarrow ClC_4H_8^*$	k_{for}	(2)
$ClC_4H_8^* \rightarrow Cl + i\text{-}C_4H_8$	k_{rev}	(3)
$ClC_4H_8^* + M \rightarrow ClC_4H_8 + M$	k_M	(4)
$ClC_4H_8^* \rightarrow HCl + C_4H_7$	k_E	(5)
$HCl \rightarrow$ quenching or loss,	k_q	(6)

in which reaction (1) is the direct abstraction reaction, reaction (2) is the formation of energetic adduct $ClC_4H_8^*$, reaction (3) is its reverse reaction, reaction (4) is the addition-stabilization reaction to form the "stable" radical adduct ClC_4H_8, and reaction (5) is the addition-elimination reaction, including the roaming path. Reactions (1) and (5) both contribute to the metathesis reaction to form HCl. According to Joalland *et al.*,[1] the addition-elimination reaction proceeds via the roaming path. Since we are probing emission of HCl, reaction (6) includes both the loss of HCl (due to diffusion and other processes) and quenching of HCl(v = 1).

Solving these differential equations yields the temporal profiles of HCl,

$$HCl(t) = [Cl]_0 \{X + \exp(-k_1 t) + Y \exp(-k_2 t) + Z \exp(-k_q t)\} \quad (7)$$

in which

$$X = (\alpha/2\kappa - \beta/2)\, [i\text{-}C_4H_8]_0 / \Gamma\, k_{for} \quad (8)$$

$$Y = (-\alpha/2\kappa - \beta/2)\, [i\text{-}C_4H_8]_0 / \Gamma\, k_{for} \quad (9)$$

$$Z = \beta\ [i\text{-}C_4H_8]_0 / \Gamma\ k_{for} \tag{10}$$

$$k_1 = (k - \kappa)/2 \tag{11}$$

$$k_2 = (k + \kappa)/2 \tag{12}$$

and

$$\Gamma = \{(k_q - k_E - k_M - k_{rev})\ (k_q - [i\text{-}C_4H_8]_0\ k_{abs} - [i\text{-}C_4H_8]_0\ k_{for}) - [i\text{-}C_4H_8]_0\ k_{for}\ k_{rev}\}/k_{for} \tag{13}$$

$$\kappa = \{(-k_E - k_{rev} - k_M + [i\text{-}C_4H_8]_0\ k_{abs} + [i\text{-}C_4H_8]_0\ k_{for})^2 + 4\ [i\text{-}C_4H_8]_0\ k_{for}\ k_{rev}\}^{1/2}$$

$$k = [i\text{-}C_4H_8]_0\ k_{for} + [i\text{-}C_4H_8]_0\ k_{abs} + k_E + k_M + k_{rev} \tag{14}$$

$$\alpha = 2\ k_q\ k_E\ k_{for} - 2\ k_E\ k_{rev}\ k_{abs} - 2\ k_E\ k_M\ k_{abs} - 2\ k_M\ k_{rev}\ k_{abs} - k_M\ k_E\ k_{for} - k_{rev}\ k_E\ k_{for} + k_{abs}\ k_E\ k_q - k_q\ [i\text{-}C_4H_8]_0\ k_{abs}^2 + k_{abs}\ k_{rev}\ k_q + k_E\ [i\text{-}C_4H_8]_0\ k_{abs}^2 + k_{rev}\ [i\text{-}C_4H_8]_0\ k_{abs}^2 + k_M\ [i\text{-}C_4H_8]_0\ k_{abs}^2 + k_{abs}\ k_M\ k_q - [i\text{-}C_4H_8]_0\ k_{for}^2\ k_E - k_{rev}^2\ k_{abs} - k_E^2\ k_{abs} - k_E^2\ k_{for} - k_M^2\ k_{abs} - k_{abs}\ k_q\ [i\text{-}C_4H_8]_0\ k_{for} + k_{abs}\ k_M\ [i\text{-}C_4H_8]_0\ k_{for} - [i\text{-}C_4H_8]_0\ k_{for}\ k_{rev}\ k_{abs} \tag{15}$$

$$\beta = k_{rev}\ k_{abs} + k_E\ k_{abs} + k_E\ k_{for} - k_q\ k_{abs} + k_M\ k_{abs} \tag{16}$$

D. Sensitivity and error analysis

The observed temporal profiles and the simulations according to fitted parameters are shown in Fig. S2−S4 for experimental sets A−C, respectively. The sensitivity analysis was performed; representative results corresponding to conditions in Fig. 2 are shown in Fig. S5. From this analysis we found that the rate coefficients k_{abs} and k_{for} are most important in affecting the rate coefficient of k_E.

We tested the sensitivity of rate coefficients k_{abs} to the fitted results. On varying k_{abs} by $\pm 2.3\times10^{-11}$ cm^3 molecule^{-1} s^{-1}, we found that k_E of sets B and C (in which roaming is more important) increased by (16±6) % at $k_{abs} = 2.3\times10^{-11}$ cm^3 molecule^{-1} s^{-1} and decreased by (16±6) % at $k_{abs} = 6.9\times10^{-11}$ cm^3 molecule^{-1} s^{-1}. Values of $\varepsilon_2/\varepsilon_1$ for sets B and C remain within uncertainty limits, with $\varepsilon_2/\varepsilon_1$ = (4.8±0.7) % at $k_{abs} = 2.3\times10^{-11}$ cm^3 molecule^{-1} s^{-1} and $\varepsilon_2/\varepsilon_1$ = (6.3±1.0) % at $k_{abs} = 6.9\times10^{-11}$ cm^3 molecule^{-1} s^{-1}.

We varied k_{for} from 1.5×10^{-10} to 5.8×10^{-10} cm^3 molecule^{-1} s^{-1}, the fitted k_E deviated from those derived with $k_{for} = 2.9\times10^{-10}$ cm^3 molecule^{-1} s^{-1} by less than 10 % in experimental set A, and less than 5 % in experimental set B. For set C with $k_{for} = 1.5\times10^{-10}$ cm^3 molecule^{-1} s^{-1}, k_E could not be fitted properly for data at P(Ar) = 1.91 and 2.99 Torr because k_{for} was too small, whereas with $k_{for} = 5.8\times10^{-10}$ cm^3 molecule^{-1} s^{-1}, k_E deviated from those derived with $k_{for} = 2.9\times10^{-10}$ cm^3 molecule^{-1} s^{-1} by less than 6 % for P(Ar) = 0.94 and 1.91 Torr, less than 37% for P(Ar) = 2.99 Torr. All deviations derived in these analysis are much smaller than the enhancement of k_E observed in experimental sets B and C as compared to those in experiments with little buffer gas.

We also tried to fit k_{abs} by setting $k_E = 0$, and found that the temporal profile could not be fitted properly, especially at higher pressure. Figure S6 compares the observed temporal profiles with the simulation according to these fitted parameters; significant deviations between observation and fitted results were observed.

We added temporal profiles of HCl (v = 1) and HCl (v = 2) to yield temporal profile of HCl (v = 1 and 2) and perform the fitting similar to those described in the main text except without consideration of vibrational branching. The parameters k_E, k_{rev}, and k_q were nearly identical to those listed in Table I. This is conceivable because the contribution of HCl (v = 2) to the total population is small. On the other hand, these results show that the consideration of vibrational distribution of HCl from abstraction and from elimination reactions does not affect the fitting of k_E.

Table S1. Experimental conditions, nascent rotational temperatures T_R for HCl (v = 1) and HCl (v = 2), average rotational energies (E_R) observed in three sets of experiments.

Set	$P_{Cl2C2O2}$ /Torr	$P_{i\text{-}C4H8}$ /Torr	P_M (M) /Torr	T_R (v = 1) /K	T_R (v = 2) /K	E_R /kJ mol^{-1}
A	0.010	0.213	0.010 (Ar)	420±40	230±220	3.9±0.5
	0.010	0.482	0.010 (Ar)	370±20	320±240	3.3±0.1
	0.011	0.687	0.011 (Ar)	360±30	370±550	3.2±0.1
	0.011	0.914	0.011 (Ar)	340±30	440±980	3.0±0.2
B	0.011	0.224	0.940 (He)	370±20	330±70	3.2±0.0
	0.011	0.225	1.910 (He)	350±20	310±80	3.0±0.0
	0.011	0.226	2.990 (He)	340±20	320±90	2.9±0.1
C	0.011	0.224	0.940 (Ar)	350±20	330±80	3.1±0.0
	0.011	0.225	1.910 (Ar)	340±30	340±60	2.8±0.1
	0.011	0.226	2.990 (Ar)	350±40	350±80	2.9±0.2

Table S2. Experimental conditions, fitted rate coefficients of elimination k_E and their associated k_{rev}, rate coefficient of quenching k_q, branching ratios for formation of HCl (v = 2) over HCl (v = 1) ϕ_2/ϕ_1 (from abstraction) with $\varepsilon_2/\varepsilon_1$ (from elimination) fixed at 0.06 in three sets of experiments.

Set	Conditions			Fitting of HCl(v = 1) and HCl(v = 2)			
	$P_{Cl2C2O2}$ /Torr	$P_{i\text{-}C4H8}$ /Torr	P_M (M) /Torr	k_E /10^5 s^{-1}	k_{rev} /10^5 s^{-1}	k_q /10^5 s^{-1}	ϕ_2/ϕ_1 (abs.)
A	0.010	0.213	0.010 (Ar)	0.5	19.8	0.6	0.02
	0.010	0.482	0.010 (Ar)	2.4	43.5	1.0	0.03
	0.011	0.687	0.011 (Ar)	2.1	63.3	1.5	0.03
	0.011	0.914	0.011 (Ar)	1.5	85.6	2.1	0.04
B	0.011	0.224	0.940 (He)	9.0	11.4	1.0	0.01
	0.011	0.225	1.910 (He)	8.8	10.9	1.1	0.01
	0.011	0.226	2.990 (He)	7.8	10.9	1.2	0.00
C	0.011	0.224	0.940 (Ar)	10.7	9.7	1.2	0.00
	0.011	0.225	1.910 (Ar)	19.6	0.0	2.0	0.00
	0.011	0.226	2.990 (Ar)	18.7	0.0	3.1	0.04

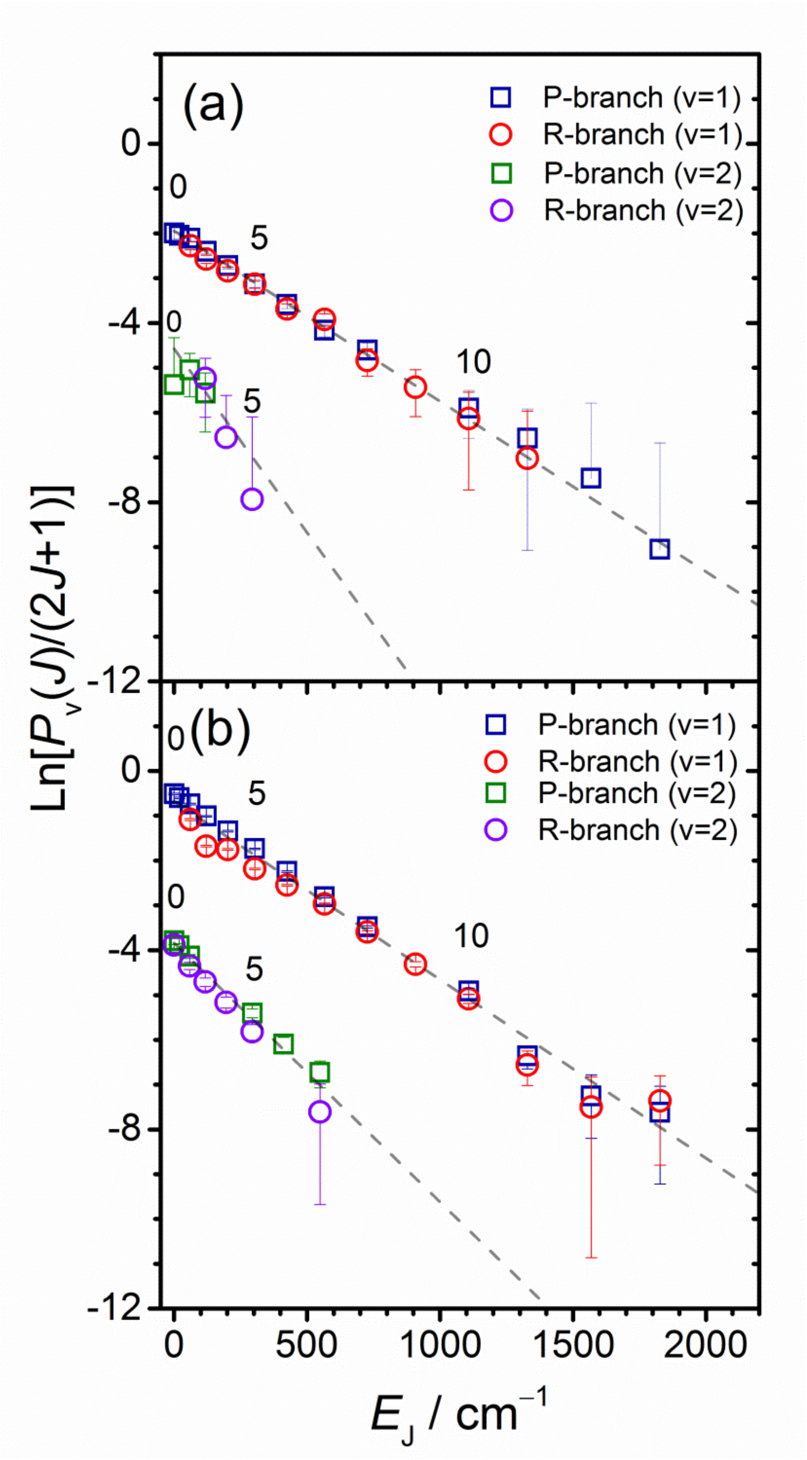

Figure S1. Semi-logarithmic plots of relative rotational populations of HCl (v = 1 and 2) 0–1 μs after photolysis. (**a**) A flowing mixture of $Cl_2C_2O_2$ (10 mTorr), *i*-C_4H_8 (213 mTorr) and Ar (10 mTorr). (**b**) A flowing mixture of $Cl_2C_2O_2$ (11 mTorr), *i*-C_4H_8 (226 mTorr) and Ar (2.99 Torr). Symbols o indicate the *R*-branch and □ the *P*-branch. Solid lines represent least-square bimodal fits.

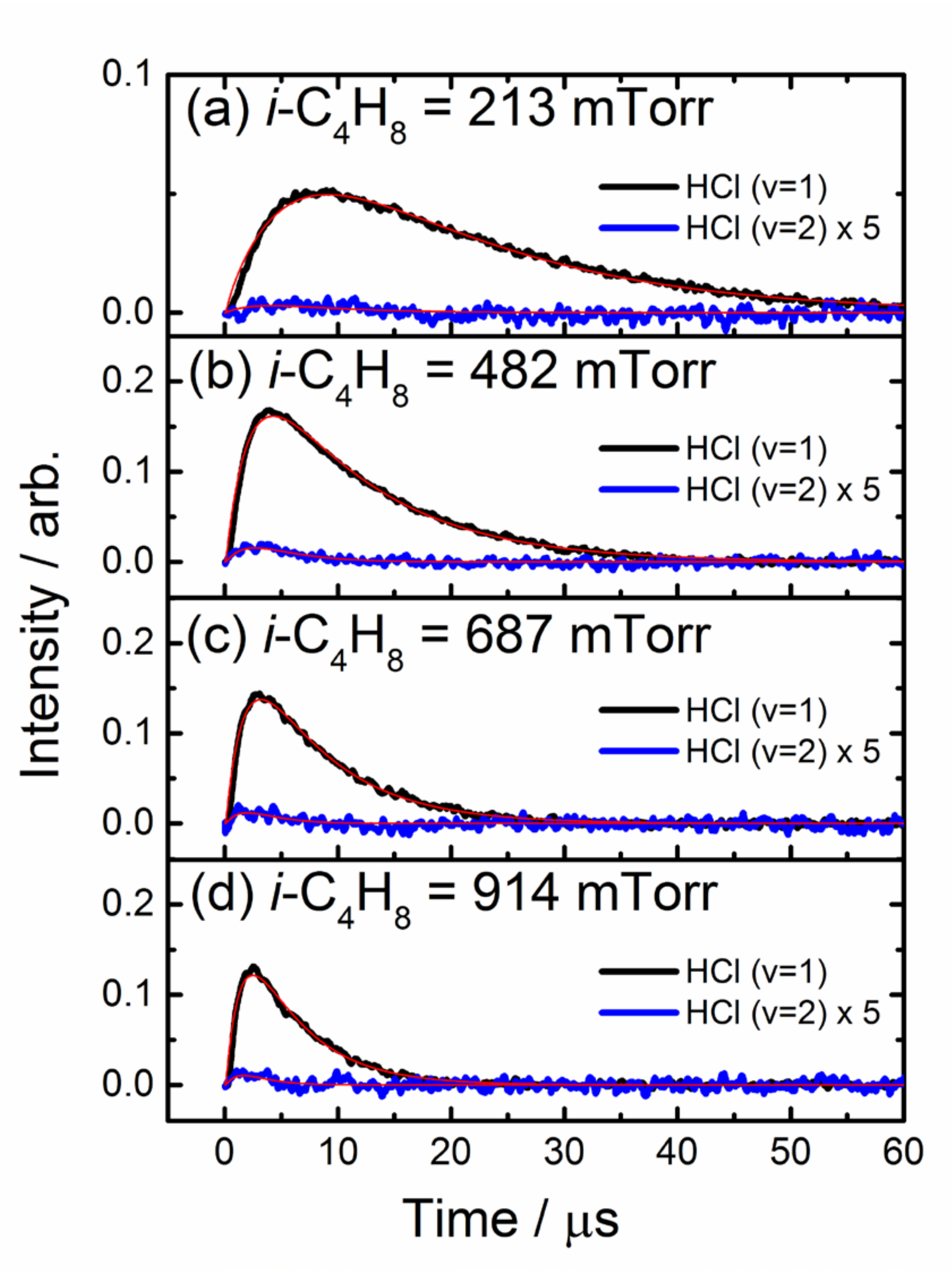

Figure S2. Temporal profiles of HCl (v = 1) and HCl (v = 2) in experimental set A with a flowing mixture of $Cl_2C_2O_2$ (10 mTorr), *i*-C_4H_8 (213−914 mTorr) and Ar (10 mTorr). The red lines represent simulated profiles according to rate coefficients listed in Table 1 of the main text.

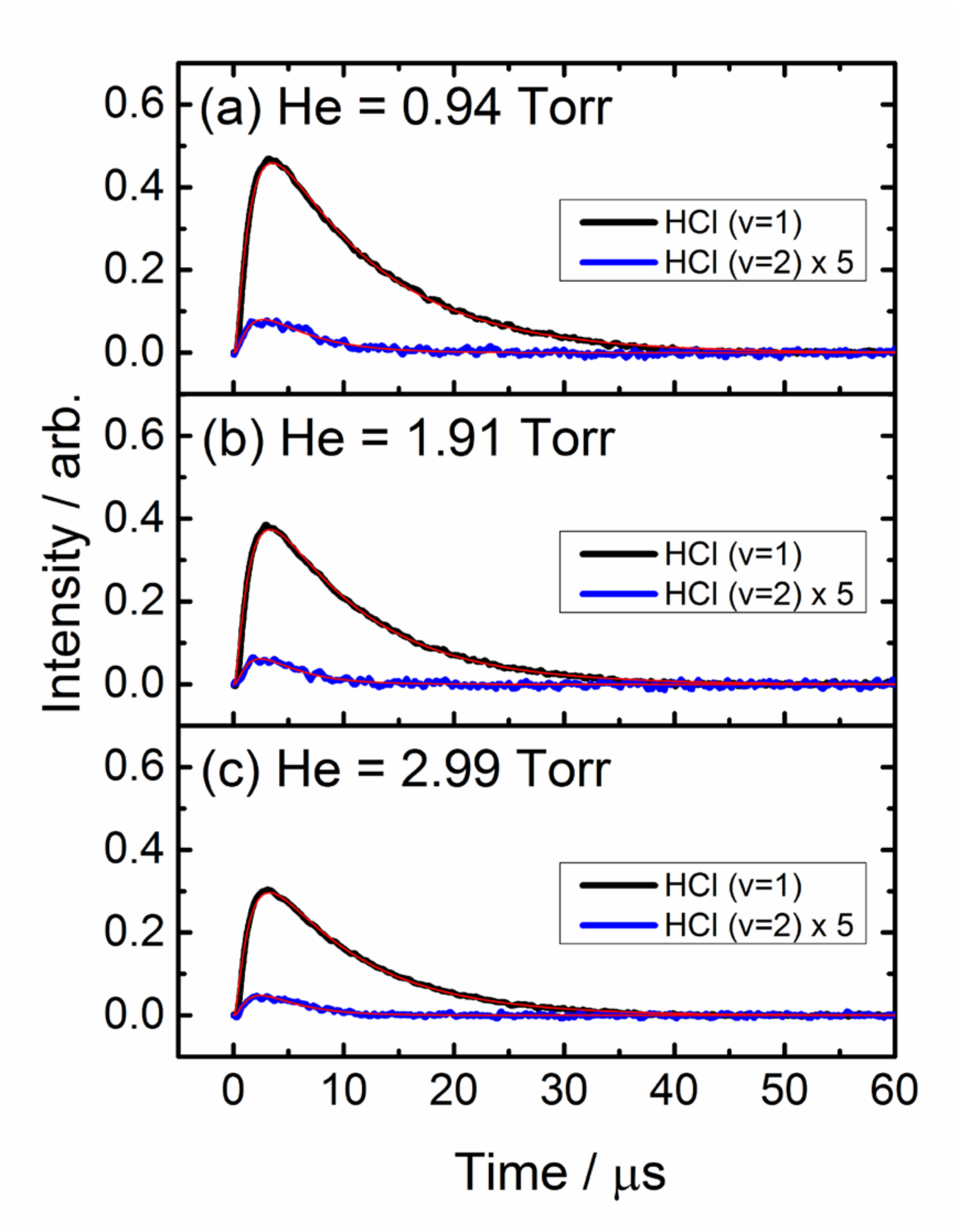

Figure S3. Temporal profiles of HCl (*v* = 1) and HCl (*v* = 2) in experimental set B with a flowing mixture of $Cl_2C_2O_2$ (10 mTorr), *i*-C_4H_8 (226 mTorr) and He (0.94−2.99 Torr). The red lines represent simulated profiles according to rate coefficients listed in Table 1 of the main text.

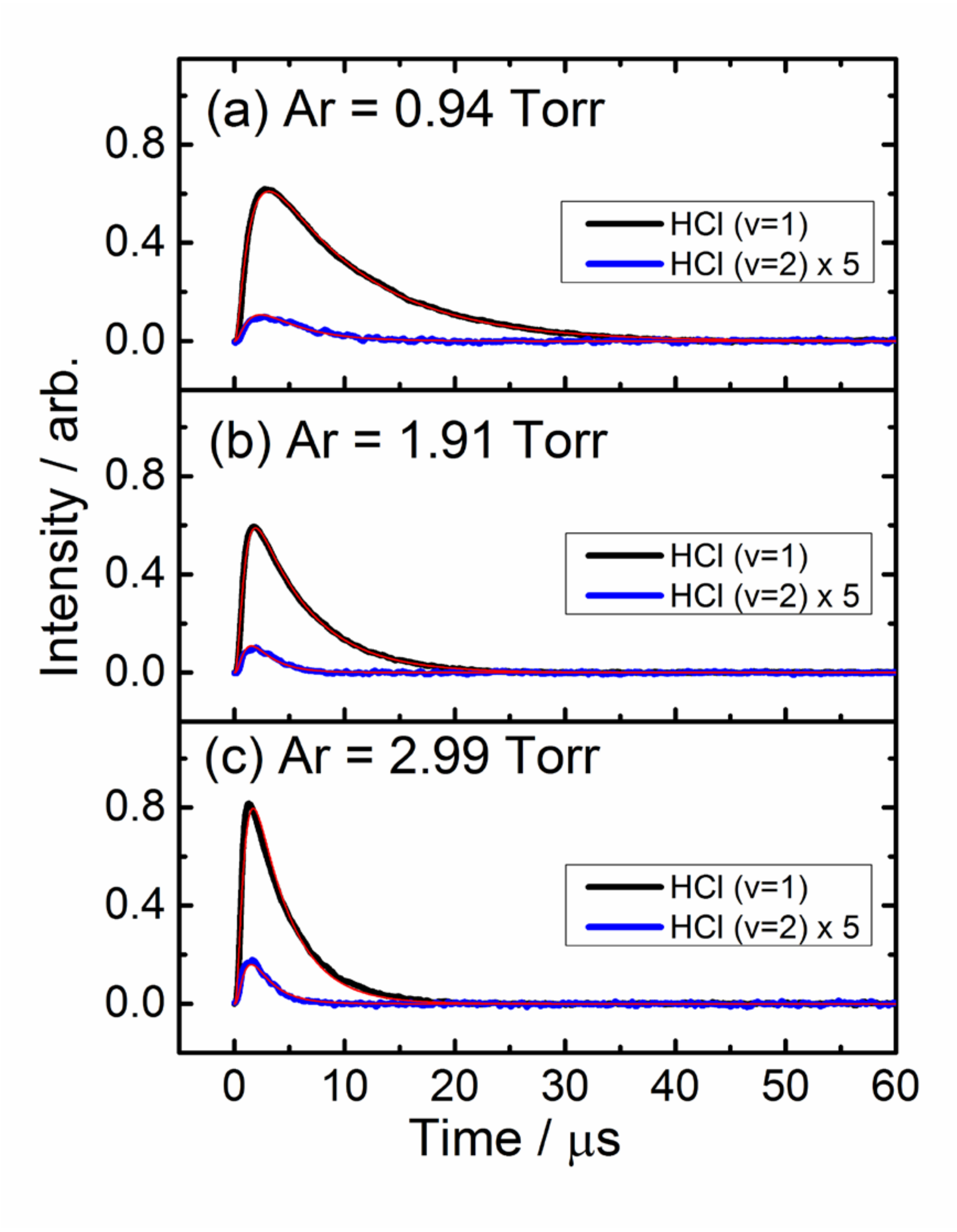

Figure 4. Temporal profiles of HCl (*v* = 1) and HCl (*v* = 2) in experimental set C with a flowing mixture of $Cl_2C_2O_2$ (10 mTorr), *i*-C_4H_8 (226 mTorr) and Ar (0.94−2.99 Torr). The red lines represent simulated profiles according to rate coefficients listed in Table 1 of the main text.

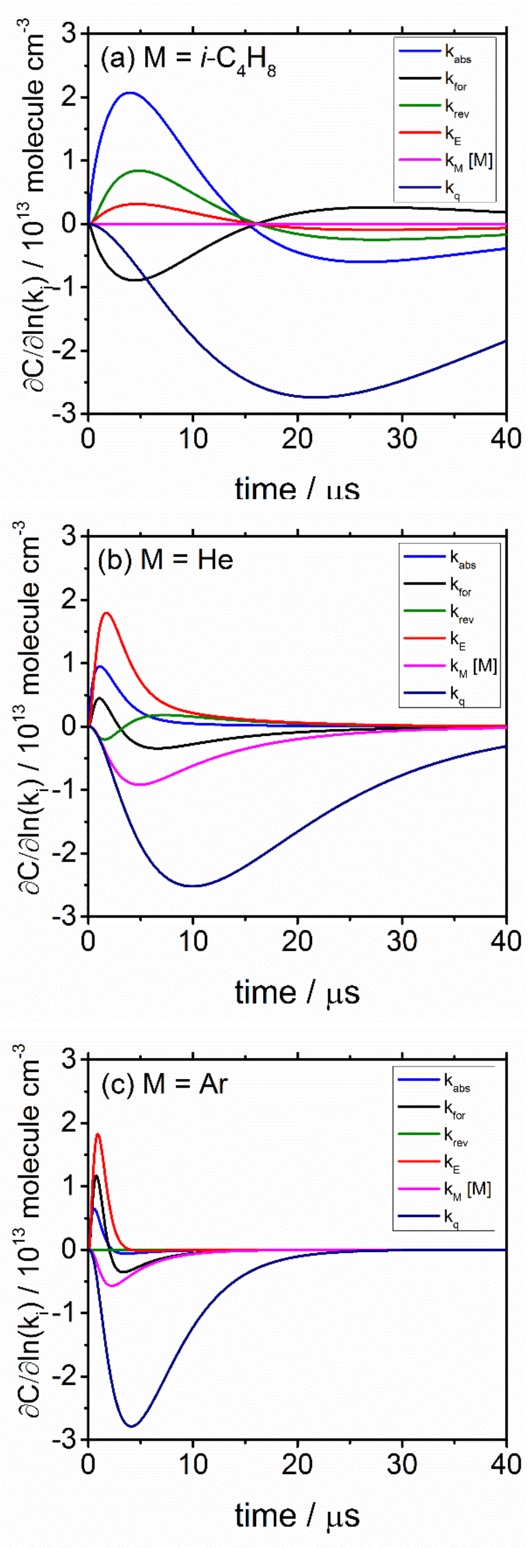

Figure 5. Sensitivity analysis. (a) A flowing mixture of $Cl_2C_2O_2$ (10 mTorr), *i*-C_4H_8 (213 mTorr) and Ar (10 mTorr). **(b)** A flowing mixture of $Cl_2C_2O_2$ (10 mTorr), *i*-C_4H_8 (226 mTorr) and He (2.99 Torr). **(c)** A flowing mixture of $Cl_2C_2O_2$ (10 mTorr), *i*-C_4H_8 (226 mTorr) and Ar (2.99 Torr). Parameters are described in the text.

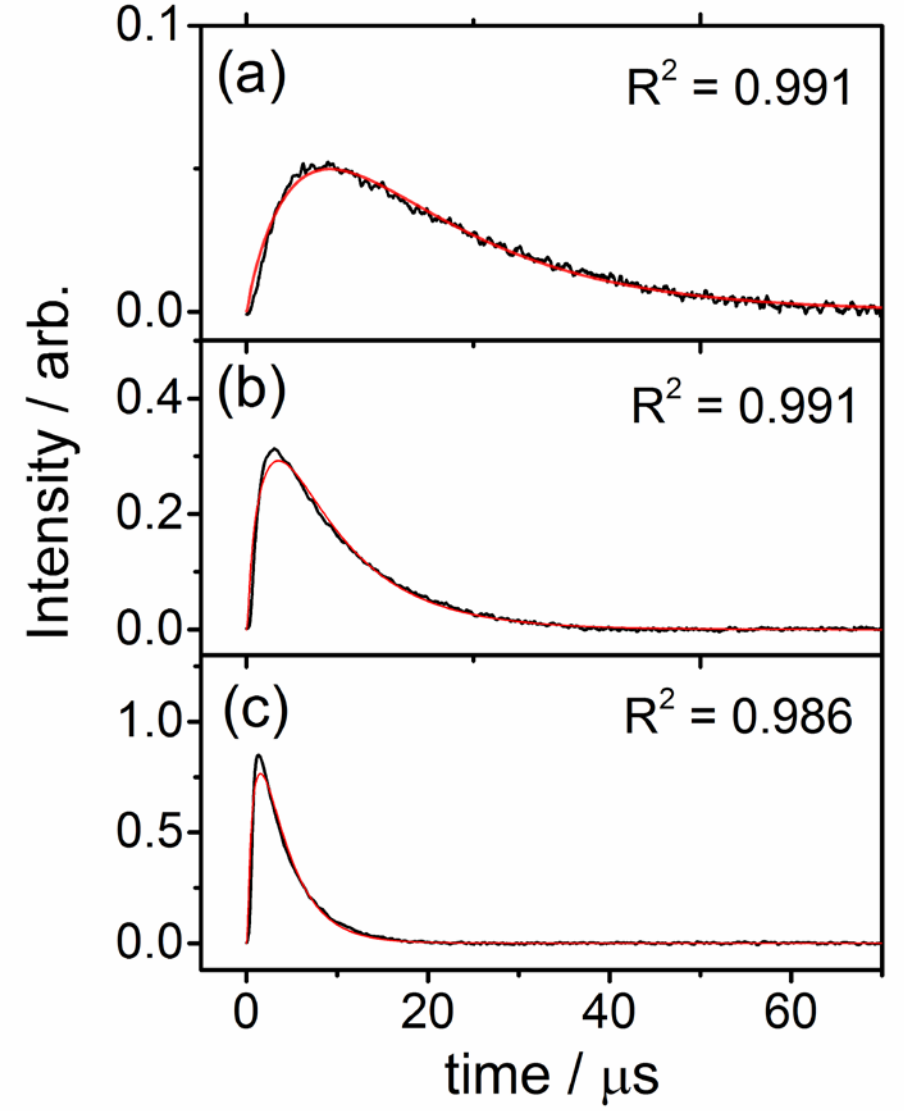

Figure 6. Comparison of observed temporal profiles of HCl (*v*) with simulations using k_E = 0. (**a)** A flowing mixture of $Cl_2C_2O_2$ (10 mTorr), *i*-C_4H_8 (213 mTorr) and Ar (10 mTorr). (**b)** A flowing mixture of $Cl_2C_2O_2$ (10 mTorr), *i*-C_4H_8 (226 mTorr) and He (2.99 Torr). (**c)** A flowing mixture of $Cl_2C_2O_2$ (10 mTorr), *i*-C_4H_8 (226 mTorr) and Ar (2.99 Torr). HCl (*v* = 1) is in black and the kinetic simulations are in red.

References

[1] Joalland, B., Shi, Y., Kamasah, A., Suits, A. G. & Mebel, A. M. Roaming Dynamics in Radical Addition–Elimination Reactions. *Nat. Commun.* **5**, 4064 (2014).

[2] Joalland, B., Camp, R. V., Shi, Y., Patel, N. & Suits, A. G. Crossed-Beam Slice Imaging of Cl Reaction Dynamics with Butene Isomers. *J. Phys. Chem. A* **117**, 7589-7594 (2013).

[3] Chu, G. *et al.* Investigation on Addition and Abstraction Channels in Cl Reactions with 1-Butene and Isobutene. *Int. J. Mass Spectrom.* **375**, 1-8 (2015).

[4] Ahmed, M., Blunt, D., Chen, D. & Suits, A. G. UV Photodissociation of Oxalyl Chloride Yields Four Fragments from One Photon Absorption. *J. Chem. Phys.* **106**, 7617-7624 (1997).

[5] Arunan, E., Setser, D. W. & Ogilvie, J. F. Vibration-Rotational Einstein Coefficients for HF/DF and HCl/DCl. *J. Chem. Phys.* **97**, 1734-1741 (1992).

[6] Preston, T. J., Dunning, G. T., Orr-Ewing, A. J. & Vázquez, S. A. Direct and Indirect Hydrogen Abstraction in Cl + Alkene Reactions. *J. Phys. Chem. A* **118**, 5595-5607 (2014).
